# Encapsulation of Plant Extracts in a Psyllium/Starch Matrix: Synthesis and Functional Properties

**DOI:** 10.3390/molecules31061026

**Published:** 2026-03-19

**Authors:** Magdalena Krystyjan, Gohar Khachatryan, Karen Khachatryan, Robert Socha, Anna Lenart-Boroń, Mariusz Witczak, Marcel Krzan, Anna Areczuk, Martyna Waśko

**Affiliations:** 1 Department of Carbohydrates Technology and Cereal Processing, Faculty of Food Technology, University of Agriculture in Krakow, Al. Mickiewicza 21, 31-120 Krakow, Poland; anna.areczuk@urk.edu.pl; 2 Department of Food Analysis and Quality Assessment, Faculty of Food Technology, University of Agriculture in Krakow, Al. Mickiewicza 21, 31-120 Krakow, Poland; gohar.khachatryan@urk.edu.pl (G.K.); robert.socha@urk.edu.pl (R.S.); 3 Laboratory of Nanotechnology and Nanomaterials, Faculty of Food Technology, University of Agriculture in Krakow, Al. Mickiewicza 21, 31-120 Krakow, Poland; karen.khachatryan@urk.edu.pl; 4 Department of Microbiology and Biomonitoring, Faculty of Agriculture and Economics, University of Agriculture in Krakow, 30-059 Krakow, Poland; anna.lenart-boron@urk.edu.pl; 5 Department of Food Industry Engineering and Instrumentation, Faculty of Food Technology, University of Agriculture in Krakow, Al. Mickiewicza 21, 31-120 Krakow, Poland; mariusz.witczak@urk.edu.pl; 6 Jerzy Haber Institute of Catalysis and Surface Chemistry, Polish Academy of Sciences, Niezapominajek Street 8, 30-239 Krakow, Poland; marcel.krzan@ikifp.edu.pl; 7 Food Technologists Scientific Circle, Carbohydrate Technology Section, University of Agriculture in Krakow, Al. Mickiewicza 21, 31-120 Krakow, Poland; martyna.wasko@student.urk.edu.pl

**Keywords:** starch/psyllium matrix, encapsulation, potato starch, psyllium, bioactive compounds, plant extracts

## Abstract

This work presents a method to encapsulate plant extracts within a binary polysaccharide carrier and to characterize the physicochemical and rheological performance of the resulting biocomposites in the context of food use. Using a starch/psyllium matrix, extracts from *Sambucus nigra* (SN), *Aronia melanocarpa* (AM), and *Echinacea purpurea* (EP) were effectively protected and incorporated through a stepwise workflow encompassing matrix preparation, encapsulation, structural verification, and functional assessment. SEM revealed a porous network containing uniformly distributed, extract-loaded spherical structures (~800–1500 nm), while FTIR supported the presence of hydrogen bonding and hydrophobic interactions that contributed to system stability. The prepared nanoemulsions showed shear-thinning (pseudoplastic) behavior, indicating favorable processing characteristics, whereas most physicochemical and bioactivity measurements were performed on lyophilized composites. The dried materials preserved extract-specific color signatures (ΔE > 5) and exhibited distinct thermal responses: AM produced a pronounced plasticizing effect (Tg reduced by >20 °C), while the incorporation of extracts generally delayed thermal degradation, consistent with polyphenol–starch interactions. Phase-transition behavior was also altered, with melting peaks suppressed for SN and AM and melting temperatures lowered for EP. Surface analysis indicated increased hydrophobicity and a reduced polar component of surface free energy, suggesting improved moisture barrier potential. Antioxidant capacity closely tracked total phenolic content (r > 0.94), with caffeic acid contributing strongly, particularly in EP-based systems. Antimicrobial activity depended on extract type (broad-spectrum for EP, selective for SN, minimal for AM), and the comparatively higher sensitivity of Gram-negative bacteria points to improved phenolic availability and membrane interactions upon encapsulation. Collectively, these results highlight the starch/psyllium matrix as a flexible platform for stabilizing plant extracts while enabling tunable functional attributes for functional food applications.

## 1. Introduction

Plant extracts constitute a valuable source of bioactive compounds used in functional foods. Depending on the type of raw material, they can provide the human body with a wide range of constituents with documented health-promoting effects, such as polyphenols, carotenoids, flavonoids, fatty acids, dietary fiber, and compounds with probiotic potential [1,2]. These compounds have been extensively studied and widely applied due to their beneficial effects on human health, including anti-inflammatory, antidiabetic, anticancer, and hypolipidemic activities, as well as their role in supporting digestive processes, blood circulation, and metabolic regulation. Regular consumption of foods rich in bioactive compounds may further contribute to strengthening the immune system and reducing the risk of developing chronic diseases, including cardiovascular diseases, diabetes, and certain types of cancer [3,4,5,6,7,8]. Chokeberry (*Aronia melanocarpa*), black elderberry (*Sambucus nigra*), and echinacea (*Echinacea purpurea*) are widely recognized as effective plant-derived immunostimulants, and their bioactive constituents may help support immune function, particularly during the autumn and winter months. Chokeberry (*A. melanocarpa*) is a rich source of polyphenols, vitamins, and minerals with strong antioxidant potential. Black elderberry (*S. nigra*) contains high levels of vitamin C and anthocyanins and has been reported to exhibit antiviral activity. Echinacea (*E. purpurea*) provides a broad spectrum of compounds with immunostimulatory, anti-inflammatory, and antiseptic properties [9,10,11,12,13]. However, the low stability of bioactive compounds and their high sensitivity to environmental factors, such as pH fluctuations, elevated temperature, oxygen presence, and light exposure, significantly limit their direct application in food products. These factors lead to reduced bioavailability of the active components and, in some cases, may also result in undesirable changes in the sensory properties of the final product [14]. Consequently, even foods directly fortified with bioactive compounds may lose a significant proportion of their functional value during technological processing and storage. For this reason, it is essential to apply technological solutions that enable the preservation of high quality in functional foods while simultaneously minimizing process invasiveness and structural modifications of the product, thereby supporting consumer acceptance. One of the most promising approaches in this regard is encapsulation, which serves as an effective strategy for the protection and controlled release of bioactive compounds [15,16]. Numerous studies confirm the validity of using encapsulation techniques to protect labile compounds from degradation, enhance their oxidative stability, mask undesirable sensory attributes, and enable efficient and controlled release of active substances [17,18]. The dynamic development of technology currently enables the encapsulation of bioactive compounds using methods such as nanoemulsion formation, freeze-drying and spray drying and the fabrication of hydrogel matrices [19]. The selection of an appropriate encapsulation method depends both on the nature of the encapsulated material and on the properties of the carrier used. In this context, natural polymers, particularly polysaccharides and proteins, are of special importance. Among the polysaccharides commonly employed in encapsulation processes are starch, pectin, gum Arabic, and alginates [14]. Their application is driven by advantages such as biodegradability, biocompatibility, non-toxicity, GRAS status, and relatively low production cost [20].

Starch, due to its gel-forming ability and ease of modification, is widely used in the food industry as a food-grade ingredient and structuring agent, and it is also increasingly explored in modern biodegradable packaging materials [20,21,22,23,24,25]. Despite its numerous advantages, starch exhibits certain limitations, including susceptibility to retrogradation, limited stability of the formed structures, and insufficient protection of hydrophobic compounds, which justifies the need for modification or reinforcement of the polysaccharide matrix [25]. Psyllium (*Plantago psyllium*), a functional biopolymer, shows strong hydrogel-forming properties and high water-binding capacity; moreover, due to the presence of arabinoxylans, it may also provide prebiotic benefits [20,26,27]. Importantly, both starch and psyllium are widely available, food-compatible materials with well-established technological functionality (e.g., thickening, stabilization, and texture modification), which supports their suitability for designing delivery/encapsulation systems intended for food applications [20,28]. From a practical perspective, their broad availability, relatively low cost, and scalability provide an additional economic rationale for selecting these carriers [23]. However, literature reports on the use of psyllium as a standalone or hybrid encapsulation matrix remain limited. Therefore, combining starch and psyllium may offer synergistic improvements in matrix structural stability and enhance the efficiency of bioactive compound encapsulation. The scarcity of comprehensive studies on encapsulating plant extracts within a starch/psyllium system indicates a significant research gap and justifies the present study.

This study developed a method for encapsulating plant extracts in a binary polysaccharide matrix and evaluated the physicochemical and rheological properties of the resulting biocomposites to assess the system’s suitability for food applications.

The present study was designed according to a logical sequence aimed at developing a functional encapsulation system with clear structure–property relationships. First, a hybrid polysaccharide carrier based on potato starch and psyllium mucilage was formulated, combining the gel-forming ability of starch with the high water-binding capacity and prebiotic potential of psyllium. Second, three botanically distinct plant extracts, *Sambucus nigra*, *Aronia melanocarpa*, and *Echinacea purpurea*, were encapsulated within this matrix via nanoemulsion formation followed by lyophilization. Third, a comprehensive analytical approach was employed to verify the success of encapsulation (SEM, FTIR) and to characterize the resulting biocomposites in terms of properties relevant to food applications: rheological behavior (processability), color (consumer acceptance), thermal stability (processing and storage stability), surface characteristics (wettability, packaging potential), and bioactivity (antioxidant and antimicrobial activity). Finally, the observed functional properties were correlated with the phytochemical composition of the extracts, particularly the profiles of phenolic acids, to provide mechanistic insights into the differences between the three systems. This integrated approach ensures that each experimental method contributes to a coherent understanding of the encapsulation system and its potential utility in functional food development.

## 2. Results and Discussion

### 2.1. The Extraction Yield

The extraction yield varied depending on the botanical origin of the raw material used (Table 1): dry elderberry fruit (DARY NATURY Sp. z o.o., Koryciny, Poland), dry chokeberry fruit (Bielsk Podlaski, Poland) and dry purple coneflower (Zioła z Doliny Bobru Grzywa Maciej, Wleń, Poland). The highest yield was obtained for *Sambucus nigra* (59.12%), followed by *Aronia melanocarpa* (53.67%), while the lowest extraction yield was recorded for *Echinacea purpurea* (42.39%).

The high extraction efficiency observed in this study results from the synergistic application of combined techniques, including ultrasound-assisted extraction and continuous stirring, performed using two solvents and under reduced temperature conditions. This approach enhances solvent penetration into the plant matrix and intensifies mass transfer, ultimately leading to increased extraction yield. According to Pascariu et al. [29], the applied extraction technique is more effective than microwave-assisted extraction (MAE) and can be considered a more advantageous method for anthocyanin recovery. Its superiority is primarily attributed to operation at lower temperatures, which reduces the risk of thermal degradation of bioactive compounds. In addition, this method allows for precise control of process parameters and ensures higher extraction efficiency due to the cavitation phenomenon, which enhances the release of active compounds from plant cells. Furthermore, the use of a water–ethanol mixture provides an extraction medium with optimized polarity, enabling the simultaneous recovery of both highly polar and less polar polyphenols. Water promotes swelling of the plant matrix and increases the accessibility of bioactive compounds, while ethanol improves their solubility and diffusion, resulting in a synergistic enhancement of extraction efficiency [29,30]. The low extraction temperature does not promote the degradation of thermosensitive compounds and therefore does not reduce extraction efficiency [31]. The higher extraction yield observed for *S. nigra* is likely related to its elevated content of compounds soluble not only in alcohol but also in water (including anthocyanins, flavonoids, and low-molecular-weight carbohydrates), which are readily transferred into the extraction medium. In contrast, the lower yield obtained for *A. melanocarpa* may result from the more compact structure of the fruits and the higher contribution of cell wall constituents, such as cellulose and lignin, which can restrict solvent penetration despite the high phenolic content of chokeberry fruits [32,33,34,35]. In contrast, the lowest extraction yield observed for *E. purpurea* is associated with its fibrous plant structure and the presence of bioactive compounds that are more strongly bound to the plant matrix, making them less susceptible to extraction under the applied conditions [9,36].

### 2.2. Rheological Characteristic of Nanoemulsions

**Figure 1:**
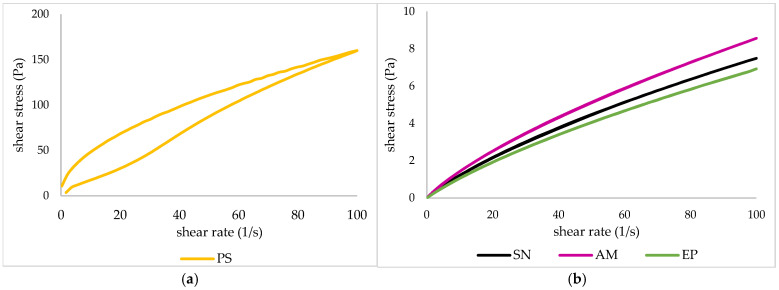
Flow curves of (**a**) starch gel and (**b**) nanoemulsions of plant extracts encapsulated in psyllium/starch matrices.

To assess the processability and structural stability of the encapsulation system prior to drying, the rheological behavior of the nanoemulsions was investigated. The flow curves (Figure 1) and rheological model parameters (Table 2) provide insight into the interactions between the polysaccharide matrix and the dispersed lipid phase containing the plant extracts.

All the tested systems exhibited pseudoplastic, shear-thinning behavior. The nanoemulsions were characterized by low shear stress values, indicating low viscosity. The parameters of the power-law model confirmed that the nanoemulsions behaved as shear-thinning fluids (n < 1) with low consistency indices (K < 1). The observed differences between the nanoemulsions and the starch gel resulted from the nanoemulsion preparation process. The homogenization process significantly influenced the physicochemical properties of the produced nanoemulsions. High shear and cavitation forces generated during homogenization resulted in a marked reduction in the size of dispersed-phase droplets. This decrease in droplet diameter increased the interfacial surface area, thereby facilitating the adsorption of stabilizing agents at the oil–water interface. Moreover, homogenization promoted the formation of a more uniform emulsion structure, which may enhance interactions between emulsion droplets and the polysaccharide matrix. In terms of bioactive compound encapsulation, homogenization played a crucial role in the efficient dispersion of hydrophobic components, leading to improved emulsion stability and enhanced immobilization efficiency of plant extracts within the polysaccharide matrix. The hysteresis loop areas of the analyzed emulsions were small and positive, indicating antithixotropic behavior associated with the reconstruction of a structure previously disrupted by shear. The presence of polysaccharides (psyllium and starch) enabled effective stabilization of the emulsions by limiting flocculation and coalescence phenomena [37]. Conventional emulsions lack thermodynamic stability due to the positive free energy generated at the interface between the oil and water phases, which is reflected in a high interfacial tension. Consequently, one of the key challenges in the food industry is to limit destabilization processes in order to extend the shelf life of final products. Therefore, stabilizing agents are incorporated into emulsion-based foods to prevent or slow down these instability mechanisms [38]. As claimed by McClements [39], oil droplets dispersed within a gelled aqueous biopolymer matrix can significantly influence the rheological behavior of the system. When these droplets are effectively integrated into the gel network, they contribute to strengthening the gel structure.

In summary, reducing droplet diameter and increasing the interfacial area through the applied technological operations facilitates the uniform dispersion and entrapment of hydrophobic components within the gel matrix. In practice, this enables the design of more stable carriers for bioactive extracts/oils, providing a more reproducible dose of active ingredients and greater resistance to destabilization over time.

### 2.3. Scanning Electron Microscopy

**Figure 2:**
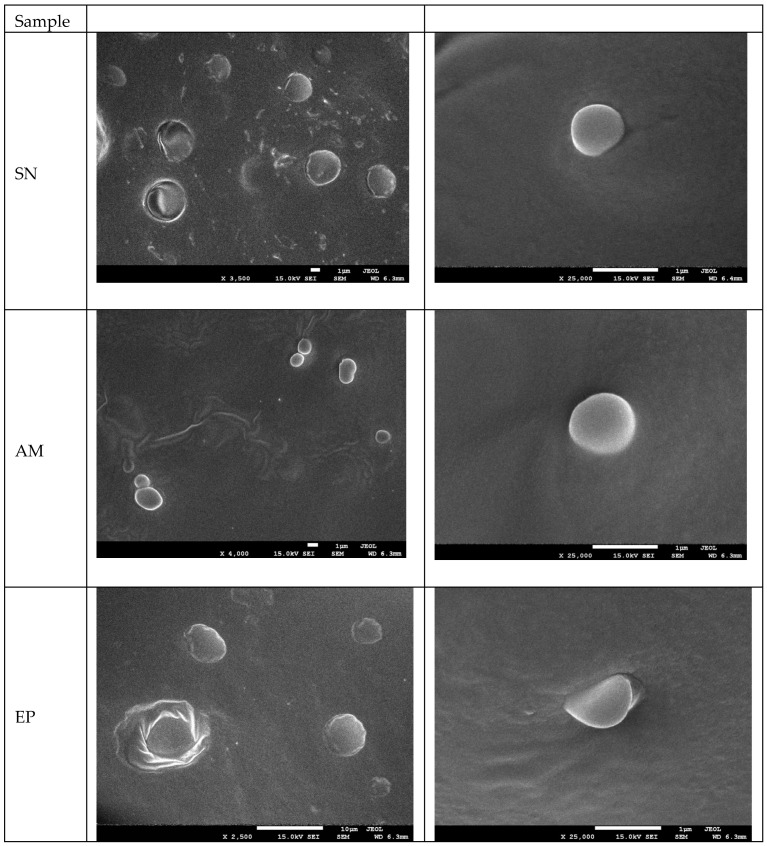
Representative scanning electron microscopy (SEM) images of the freeze-dried polysaccharide matrices: (SN) matrix containing *Sambucus nigra* extract; (AM) matrix containing *Aronia melanocarpa* extract; (EP) matrix containing *Echinacea purpurea* extract, at magnifications of 2500×, 3500×, 4000× and 25,000×, respectively.

Having established the rheological stability of the nanoemulsions, the morphology of the freeze-dried biocomposites was examined by SEM to verify the successful incorporation of the extract-containing droplets within the polysaccharide network. Scanning electron microscopy (SEM) was used to visualize the morphology of the obtained nanocomposites (Figure 2). The SEM images reveal that all analyzed samples possess a heterogeneous, porous, sponge-like 3D structure, which is characteristic of freeze-dried hydrocolloid materials based on starch and psyllium mucilage [20,25]. The formation of such a network is attributed to the sublimation of ice crystals during the lyophilization process, leaving behind cavities and creating a scaffold that immobilizes the active ingredients.

The microstructures of the composites enriched with nanoemulsions containing plant extracts (SN, AM, EP) showed distinct morphological changes. The presence of spherical and oval microstructures embedded within the polymer matrix or adhering to the surface of the pore walls was observed. These spherical structures ranged in size from approximately 800 to 1500 nm, corresponding to the oil droplets and encapsulated bioactive compounds stabilized by the starch–psyllium system. The homogenization process, utilizing ultrasonic treatment, facilitated the dispersion of the lipid phase (grape seed oil) and plant extracts, preventing significant coalescence of the droplets during the gelation and drying phases. The visible roughness on the pore surfaces in samples SN, AM, and EP suggests a successful incorporation of the hydrophobic core material into the hydrophilic polysaccharide network. Similar morphological features, where active compounds are entrapped within the pore walls of a biopolymer matrix, have been reported in previous studies on starch- and chitosan-based carriers [14,37,40]. The structural integrity observed in the SEM micrographs correlates with the rheological stability of the emulsions prior to drying, confirming the compatibility of the psyllium/starch matrix with the applied plant extracts.

The morphological observations confirm that the porous starch/psyllium matrix acts as an effective scaffold for immobilizing the nanoemulsions, preventing coalescence during freeze-drying and ensuring uniform distribution of the bioactive components. This structural integrity is essential for the subsequent functional properties of the composites, including their thermal behavior, surface characteristics, and bioactivity.

### 2.4. Structure of the Matrices

**Figure 3:**
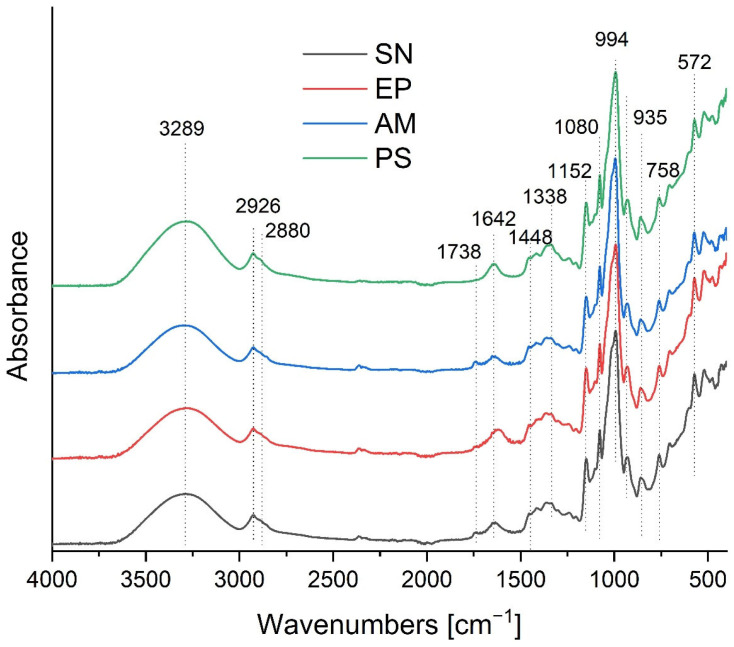
FTIR spectra of the control psyllium/starch matrix (PS) and the biocomposites enriched with encapsulated plant extracts: *Sambucus nigra* (SN), *Aronia melanocarpa* (AM), and *Echinacea purpurea* (EP). The dashed lines indicate the characteristic wavenumbers discussed in the text, highlighting the spectral changes associated with the incorporation of the lipid phase and bioactive compounds.

Fourier-transform infrared spectroscopy (FTIR) was employed to identify the functional groups present in the carrier matrix and to evaluate the interactions between the polysaccharide components and the encapsulated plant extracts. The FTIR spectra of the control sample (PS) and the composites containing elderberry (SN), chokeberry (AM), and purple coneflower (EP) extracts are shown in Figure 3.

All spectra exhibited characteristic bands typical for polysaccharide backbones (starch and psyllium). A broad and intense band centered around 3289 cm^−1^ corresponds to the O–H stretching vibrations of hydroxyl groups. This band is associated with the formation of inter- and intramolecular hydrogen bonds within the starch and psyllium chains, as well as interactions with water molecules [14,16]. The bands in the region of 2926 cm^−1^ and 2880 cm^−1^ are attributed to C–H stretching vibrations (asymmetric and symmetric) of methylene (–CH_2_–) and methyl (–CH_3_) groups. It is worth noting that the intensity of these peaks increased in the samples containing plant extracts (SN, AM, EP) compared to the control (PS). This enhancement is likely due to the contribution of aliphatic chains from the fatty acids present in the grape seed oil used as the carrier for the extracts, as well as lipophilic compounds from the extracts themselves [16,40].

A significant spectral difference between the samples enriched with nanoemulsions (SN, AM, EP) and the control matrix (PS) was observed in the carbonyl region, where a new, distinct band appeared at a wavenumber of approximately 1738 cm^−1^. This signal originates from the stretching vibrations of C=O bonds in ester groups and serves as direct evidence of the presence of lipid phase components within the composite structure, specifically triglycerides derived from the grape seed oil used as a carrier [14,16,40]. The increase in the intensity of this band may also be potentiated by the presence of organic acids and esterified phenolic compounds contained within the introduced plant extracts, confirming the effective incorporation of the active material into the polysaccharide matrix [19,26].

The band at 1642 cm^−1^ is assigned to the bending vibrations of adsorbed water molecules (δ O–H) and may also overlap with the vibrations of carboxylate groups naturally present in the psyllium mucilage arabinoxylans. The region between 1200 cm^−1^ and 900 cm^−1^, known as the “fingerprint region” for polysaccharides, showed intense bands at 1152 cm^−1^ and 1080 cm^−1^, and a sharp peak at 994 cm^−1^ with a shoulder at 935 cm^−1^. These are characteristic of C–O–C stretching vibrations of the glycosidic linkages and C–O/C–C stretching in the pyranose rings of starch and psyllium [20,37]. The preservation of these bands in the composite spectra indicates that the chemical structure of the polysaccharide matrix remained stable after the incorporation of the extracts. The slight shifts in wavenumbers and changes in peak intensities suggest the formation of hydrogen bonds and van der Waals interactions between the biopolymer matrix and the encapsulated bioactive compounds [19].

The observed spectral changes, particularly the appearance of the carbonyl band at ~1738 cm^−1^ and the increased intensity of C–H stretching vibrations, demonstrate not only the successful incorporation of the grape seed oil and lipophilic extract components but also the formation of physical interactions (hydrogen bonds, van der Waals forces) between the polysaccharide matrix and the encapsulated material. These interactions are crucial for the stability of the system, as they limit phase separation and promote the retention of bioactive compounds within the matrix. The preservation of the characteristic polysaccharide fingerprint region (1200–900 cm^−1^) indicates that the chemical structure of the carrier remains intact, while the slight shifts in band positions suggest molecular-level rearrangements that may influence the functional properties of the composites, such as their thermal behavior and surface wettability.

### 2.5. Color Measurement

Table 3 summarizes the color parameters of the obtained biocomposites and the freeze-dried starch gel used as the base material for the preparation of these matrices. Color characteristics were described using three fundamental parameters: lightness (L*), hue angle (h*), and chroma (C*). The visual appearance of the biocomposites, quantified by CIELAB color parameters (Table 3), is directly related to the phytochemical composition of the encapsulated extracts and provides information on the retention of native pigments after processing.

All the obtained matrices exhibited high color lightness; however, statistically significant differences were observed among the individual samples (*p* < 0.05). The base matrix (PS) showed the highest L* value (97.34), indicating a color close to pure white. The lightness of the remaining matrices was strongly influenced by the presence of plant extracts, with darker extracts resulting in lower L* values and darker matrix appearance.

Analysis of the a* and b* color coordinates revealed that the PS matrix displayed a slight predominance of the yellow component over the blue one (b*), while maintaining a near balance between the red and green components (a* ≈ 0). In contrast, the matrix containing black elderberry extract (SN) showed a pronounced dominance of the red and yellow components, indicating a shift toward warmer color tones. A similar tendency was observed for the matrix enriched with chokeberry extract (AM), although the intensity of these components was approximately twofold lower than in the SN sample. These trends were further confirmed by the hue angle values, which describe the qualitative aspect of color. The hue values for the SN and AM matrices corresponded to light red shades, whereas the matrix containing purple coneflower extract (EP) exhibited a shift toward light green hues. Color saturation (C*) was highest for the SN and EP matrices, indicating greater color intensity and purity, while the AM sample was characterized by approximately twofold lower color saturation.

The dominant effect of black elderberry and chokeberry fruit extracts on the color parameters of polysaccharide matrices results primarily from their high anthocyanin content, which constitutes the main group of pigments responsible for the red–violet and purple hues of these raw materials. In the case of black elderberry (*S. nigra*), cyanidin derivatives—particularly cyanidin-3-glucoside and cyanidin-3-sambubioside—play a key role, contributing to the intense, dark coloration of the extracts and their pronounced effect on decreasing the L* parameter while increasing the a* and b* values in the CIELAB system [12,13,41,42]. Black chokeberry (*A. melanocarpa*) fruits are characterized by one of the highest anthocyanin contents among berry fruits, with cyanidin-3-galactoside, cyanidin-3-arabinoside, and cyanidin-3-glucoside being the predominant compounds [11,43,44]. Despite the high concentration of these pigments, the color intensity of chokeberry extracts was lower than that of black elderberry, which may result from differences in the anthocyanin profile, their stability, and their interactions with the polysaccharide matrix. As demonstrated in the studies by Klisurova et al. [45], phenolic compounds present in chokeberry, including proanthocyanidins, hydroxycinnamic acids, and epicatechin, can act as effective copigments. Their presence promotes the stabilization of anthocyanin pigments, which explains the relatively high color stability of chokeberry compared with other berry fruits containing mainly non-acylated anthocyanins [45]. At the same time, the stability of systems based on copigmentation phenomena remains sensitive to external factors. It has been shown that both increased temperature and the presence of ethanol weaken copigmentation interactions. Even a small, five-percent proportion of alcohol used as a cosolvent can significantly reduce the strength of interactions between anthocyanins and chlorogenic acid. In addition, elevated temperature favors the dissociation of copigmentation complexes and the conversion of anthocyanins into colorless forms, which ultimately results in a loss of color intensity [46]. In contrast, the color of extracts from purple coneflower (*E. purpurea*) flowers is considerably less intense and results from a more complex phytochemical composition. Besides small amounts of anthocyanins present in the purple parts, flavonoids (including quercetin and kaempferol derivatives) and chlorophylls—particularly in the green parts of the raw material—play an important role in color formation [9]. Such a pigment profile favors lighter shades and a shift in the hue angle toward green or light red tones, which is reflected in higher L* values and different a* and b* coordinates. The observed differences in color parameters among matrices containing various plant extracts are therefore directly related to the profile and concentration of color compounds, as well as their susceptibility to degradation and interactions with the polysaccharide matrix. These results confirm that encapsulation within a starch/psyllium system enables effective immobilization of plant pigments while preserving the characteristic color features associated with the origin of the applied extract. Based on the color parameters, the total color difference (ΔE) between matrices containing plant extracts and the base matrix was calculated. For all three samples, ΔE values exceeded 5, indicating that the color differences between samples are clearly perceptible to the naked eye [47], which is of practical relevance for food applications where visual appeal influences consumer acceptance. The ability of the starch/psyllium matrix to preserve the characteristic colors of the plant extracts, ranging from the deep red of elderberry to the lighter greenish hues of Echinacea, demonstrates its suitability as a carrier for natural colorants in functional food products.

### 2.6. Thermal Properties of Nanoemulsions

The results of the DSC analysis of the tested samples are presented in Table 4 and Table 5.

**Figure 4:**
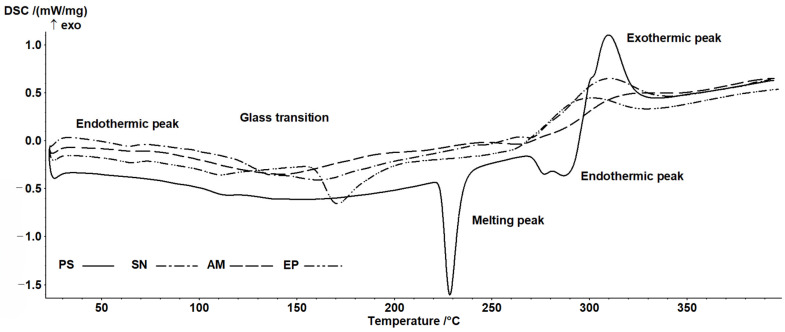
Example thermograms of the examined samples of plant extracts encapsulated in psyllium/starch matrices. SN—biocomposites with *Sambucus nigra* (elderberry) extract; AM—biocomposites with *Aronia melanocarpa* (chokeberry fruit) extract; EP—biocomposites with *Echinacea purpurea* (purple coneflower) extract; PS—freeze-dried 4% potato starch gel.

The presence of several characteristic transitions was observed. The first (endothermic) transition suggests the gelatinization of small amounts of starch, which may be related to its retrogradation or incomplete gelatinization during the production of nanocomposites (enthalpy in the range of 0.1–2.4 J/g). The second transition can be associated with the glass transition phenomenon (Table 5). As shown in Figure 4, this transition has a complex character.

On the one hand, this behavior may be related to the sample’s thermal history and relaxation phenomena, while on the other hand, it may result from interactions between the main components—starch and the plant extracts. The transition occurs within the temperature range typical for potato starch at low water activity levels [48,49,50]. The addition of the extracts resulted in a decrease in Tg, with the greatest reduction observed after the incorporation of chokeberry extract. This may be related to the relatively high content of low-molecular-weight sugars, which significantly influence glass transition temperatures. The observed differences between the samples indicate that the composition of the extracts affects the glass transition temperature. Another transition is associated with the melting of crystalline structures (Table 4). Distinct transitions were observed only for the PS and EP samples. Similarly to the glass transition, these samples exhibited comparable behavior. In the two remaining samples, although the melting phenomenon can be observed (Figure 4), it appears broadened and lacks clearly defined characteristic parameters. This suggests that the presence of chokeberry and elderberry extracts may inhibit the crystallization process during nanocomposite formation. It should also be noted that this transition showed relatively high variability, and despite noticeable differences in mean values, no statistically significant differences were found. The final peaks may be associated with the thermal degradation of polymers present in the nanocomposite composition. According to Mathew et al. [51], an exothermic peak beginning at around 250 °C in starch-based films can be attributed to polymer degradation, including dehydration of saccharide rings and depolymerization processes. Similar results were reported by Woszczak et al. [52] for starch- and chitosan-based samples. Although the variability of the values in this case is not large, a clear change in the degradation profile can be observed after the incorporation of the extracts, indicating greater stability of the samples containing the extracts.

The observed differences in glass transition temperature (Tg) between samples can be attributed to the plasticizing effect of low-molecular-weight compounds present in the extracts, such as sugars and organic acids. The most pronounced Tg reduction was observed for the chokeberry-containing composite (AM), which correlates with the relatively high content of simple sugars in Aronia fruits. This plasticizing effect increases the molecular mobility of the polysaccharide chains, which may facilitate the relaxation of the matrix and influence its mechanical properties.

The absence of clear melting endotherms for the SN and AM samples suggests that the presence of elderberry and chokeberry extracts inhibits starch recrystallization during freeze-drying. This is consistent with the known ability of polyphenols to interact with starch chains via hydrogen bonding, thereby disrupting the alignment required for crystal formation. The exothermic degradation peaks, shifted to higher temperatures in the extract-containing samples compared to the control (PS), indicate enhanced thermal stability, likely due to the antioxidant activity of the phenolic compounds, which may scavenge free radicals and delay polymer decomposition.

### 2.7. Wettability and Free Surface Energy

**Figure 5:**
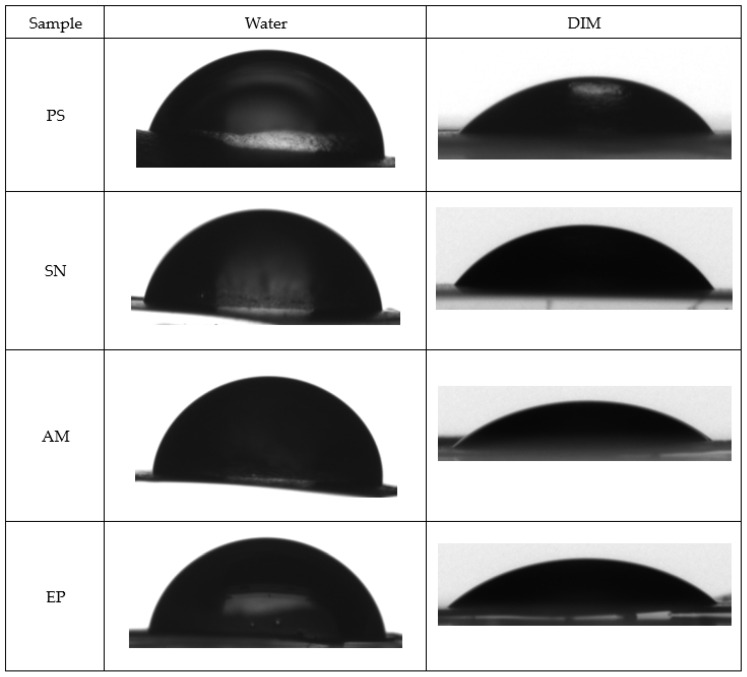
Water and diiodomethane contact angles of the starch control matrix (PS) and nanocomposites containing plant extracts: *Sambucus nigra* (SN), *Aronia melanocarpa* (AM), and *Echinacea purpurea* (EP).

The surface properties of the biocomposites, including wettability and surface free energy (Figure 5; Table 6 and Table 7), are critical for their potential application, as they influence moisture barrier performance and adhesion to food surfaces. The wettability of the obtained films was evaluated using water and diiodomethane as probe liquids. As presented in Figure 5 and Table 6, all modified samples exhibited altered wetting behavior compared to the base starch gel.

The incorporation of plant extracts markedly modified the interfacial properties of the obtained nanocomposites, influencing both their wettability and surface free energy (SFE). The water contact angles ranged from 81.9° (EP) to 90.1° (SN) (Figure 5; Table 6), positioning the films within the range of weakly hydrophilic to borderline hydrophobic surfaces. An increase relative to the starch control (PS, 84.4°) was observed for SN and AM composites, indicating that the addition of extract-rich nanoemulsions reduces the availability of hydrophilic functionalities at the material surface. The EP composite, in contrast, exhibited a slightly lower water contact angle than PS, which may reflect the higher polarity of phenolic constituents characteristic of *Echinacea purpurea*.

A similar pattern was found for diiodomethane, where contact angles varied between 40.0° (AM) and 50.3° (SN). The highest DIM angle obtained for SN suggests an enhanced role of dispersive interactions at the interface, while the AM sample—with the lowest DIM value—exhibits a more energetically favorable interaction with non-polar liquids. Comparable modifications in surface wettability upon incorporation of natural additives have been reported for polysaccharide-based systems containing waxes, oils, propolis, or nano- and microstructures, where the reorganization or partial masking of hydrophilic groups leads to decreased wettability [14,40].

The Owens–Wendt analysis confirmed these trends. The total surface free energy of the films ranged from 38.12 to 44.63 mN/m (Table 7). All extract-containing composites exhibited a markedly reduced polar component compared with the control (PS: 1.91 mN/m), with values as low as 0.74 mN/m for AM. Such a strong suppression of the polar fraction indicates reduced hydrogen-bonding capability at the surface and is consistent with the migration of lipophilic constituents (grape seed oil, extract-derived terpenoids, esters, phenolics) toward the air–solid interface during homogenisation, gelation, and freeze-drying. This surface rearrangement likely results in partial coverage or orientation changes in hydroxyl-rich starch and psyllium domains. Consequently, dispersive forces became the dominant contributors to total SFE, with the highest dispersive energy recorded for AM (43.89 mN/m), correlating with its relatively low diiodomethane contact angle.

Among the modified formulations, AM showed the highest total SFE (44.63 mN/m), whereas EP retained the highest polar contribution (3.14 mN/m), suggesting less extensive masking or reorientation of surface hydroxyl groups in this system. These differences reflect not only the physicochemical composition of the extracts but also their propensity for interfacial migration and molecular packing within the matrix. Extract-dependent variations in surface polarity directly agree with the observed water contact angles, confirming that the plant-derived components modulate the distribution and accessibility of hydrophilic and hydrophobic domains at the material surface.

Overall, the extract-induced rearrangement of surface chemistry—manifested as increased hydrophobicity, reduced polar interactions, and dominance of dispersive forces—highlights the important functional implications of encapsulation. Such surface characteristics are typically associated with reduced water sensitivity, improved moisture barrier performance, and enhanced stability during storage. These properties are advantageous for potential applications of the analyzed composites as active or functional packaging materials, aligning with previously reported behavior of polysaccharide-based bionanocomposites containing lipophilic or nanostructured additives [40].

### 2.8. Polyphenol Content and Antioxidant Activity

The bioactivity of the biocomposites, central to their potential as functional food ingredients, was evaluated by determining the phenolic content and antioxidant activity (Table 8). These results were then correlated with the detailed phytochemical profiles obtained by HPLC (Table 9) to identify the specific compounds responsible for the observed effects. In the analyzed biocomposites, enriched with elderberry, chokeberry and Echinacea extract (SN, AM, EP, respectively), the presence of phenolic compounds was identified using the spectrophotometric method (i.e., Folin–Ciocalteu’s assay), with the results expressed as mg of gallic acid per 100 g of sample. The total phenolic content (TPC) ranged from 261.27 to 921.74 mg/100 g, reaching the highest value for EP and the lowest for AM. Lyophilized potato starch (PS), used as the base (reference) for the analyzed samples, did not contain substances of a polyphenolic nature (Table 8). The obtained results were confirmed by the values of total flavonoid content (TFC) determined spectrophotometrically, which ranged from 1 to 6.27 mg/100 g of the tested product and were expressed as quercetin equivalents. Analogously to the TPC values, the EP biocomposite was characterized by the highest flavonoid content, while the chokeberry biocomposite was the poorest in this respect. When comparing the absolute values of both analyzed parameters, it can be observed that the spectrophotometrically determined flavonoid content constitutes only a small amount of the total polyphenol content; moreover, flavonoid levels in the analyzed samples were three, or even four, orders of magnitude lower than those of polyphenols. It is also important to note that the abovementioned methods, based on chemical reactions with polyphenols, are poorly selective, as the other non-phenolic substances with reducing properties may also lead to an overestimation of the results. Despite this limitation, a high Pearson correlation coefficient (r = 0.9381) was observed between these parameters.

The studied biocomposites were also characterized by a significant antioxidant (antiradical) activity against an ABTS radical cation and a DPPH radical. The values of antioxidant activity, measured in the reactions with these radicals, were expressed as mM Trolox equivalents (standard) per 100 g of sample and ranged from 2.209 to 6.517 mM/100 g for DPPH assay and from 3.343 to 9.308 mM/100 g for ABTS assay, respectively. Analogously to the TPC values, the composite with echinacea extract exhibited the highest antioxidant activity, while the AM composites showed the lowest values. The high and positive values of Pearson correlation coefficients were observed between the TPC values and the results of DPPH assay (r = 0.9411), as well as ABTS assay (r = 0.9744), respectively. This fact indicates that phenolic compounds present in the analyzed samples are responsible for the antioxidant properties of the studied samples. The results obtained using a FRAP method proved the reducing capacity of the studied extracts toward transition metal ions (Fe^3+^). The highest reducing activity, expressed as mM of Fe^3+^ ions, was observed for EP (15.296 mM), while the lowest was found for AM ones (4.370 mM). The high and positive values of Pearson correlation coefficients were found between a FRAP method and Folin method (r = 0.9950), as well as between FRAP assay and antioxidant activity measured using DPPH (r = 0.9702) and ABTS assays (r = 0.9470), respectively. This fact indicates a significant contribution of phenolic compounds to reducing properties of the analyzed samples and also confirms their health-promoting properties, characterized by both the antioxidant (antiradical) and reducing activities.

Potato starch, used as the reference base in this study, exhibited a very low reducing capacity, observed at the level of 0.644 mM, which is most likely due to the presence of small amounts of starch’s impurities, such as, e.g., short-chain maltodextrins, which possess measurable reducing properties against transition metals ions.

The presence of phenolic acids that occur mainly in the bound forms was detected in the analyzed biocomposites. Free phenolic acids (i.e., gallic and protocatechuic acids; analyzed before an alkaline hydrolysis) were detected only in the elderberry fruit composites (SN). The content of gallic acid, calculated in mg per 100 g of sample, was 0.525 mg, while that of protocatechuic acid was 0.711 mg. None of the hydroxycinnamic acids occurring in the free form were identified in the analyzed samples. As a result of the applied alkaline hydrolysis, the release of hydroxycinnamic acids (i.e., caffeic, p-coumaric, and ferulic acids) from their bound forms was observed, and an increase in the level of protocatechuic acid from 0.711 to 1.063 mg per 100 g was also noted in the case of SN. The phenolic acid present in the highest amount (and present in the bound forms) in most of the analyzed samples was caffeic acid, with level ranging from 0.782 to 49.49 mg/100 g. A very high content of that acid in EP explains the highest antioxidant and reducing values of this product among the samples under study (Table 9). High values of Pearson correlation coefficients were found between the content of this phenolic acid and antioxidant activity against the DPPH radical (r = 0.9755) as well as the FRAP method (r = 0.8931). These correlations, when analyzed using extracts not subjected to hydrolysis, most likely result from the presence of bound forms of caffeic acid with quinic acid, i.e., chlorogenic acids, in the analyzed samples.

The second most abundant phenolic isolated after an alkaline hydrolysis was *p*-coumaric acid, the content of which ranged from 0.324 mg (for AM) to 5.111 mg/100 g (for SN), respectively. This compound largely contributes to the antioxidant and reducing activity of SN due to its relatively high level in this sample. Contrary to caffeic acid, *p*-coumaric acid is not representative for the TPC and antioxidant activities of the remaining samples, since its contents did not correlate significantly with antioxidant and reducing activity values within the analyzed samples. In addition, the presence of ferulic acid was identified in the EP biocomposite at a level of 2.522 mg/100 g, providing an additional contribution to the antioxidant and reducing properties of this product. Similarly, caffeic acid, p-coumaric, and ferulic acid occur in the analyzed samples as their bound forms, most likely as chlorogenic acids (i.e., p-coumaroylquinic and feruloylquinic acids).

Among identified hydroxybenzoic acids, the release of protocatechuic acid from bound forms was observed in the AM biocomposite at an amount of 1.181 mg/100 g. The obtained results confirm that the health-promoting properties (i.e., reducing and antioxidant activities) of the analyzed samples are largely shaped by the presence of phenolic acids, including hydroxybenzoic and hydroxycinnamic acids derivatives. The applied chromatographic analysis did not confirm the presence of flavonoids or their derivatives in the analyzed samples at levels exceeding the detection limit.

The exceptionally high correlation coefficients between total phenolic content (TPC) and antioxidant activity measured by DPPH (r = 0.9411), ABTS (r = 0.9744), and FRAP (r = 0.9950) provide quantitative evidence that phenolic compounds are the primary contributors to the antioxidant properties of the biocomposites. The chromatographic analysis (Table 9) further refines this understanding by identifying caffeic acid as the dominant phenolic compound in the most active sample (EP, 49.49 mg/100 g), with a strong correlation between its content and both DPPH scavenging (r = 0.9755) and ferric reducing capacity (r = 0.8931). This confirms that caffeic acid and its derivatives (e.g., chlorogenic acids) are key bioactive agents in the echinacea composite.

The predominance of bound phenolic acids (released only after alkaline hydrolysis) in all samples indicates that the encapsulation process preserves the native esterified forms present in the plant materials. This is significant because bound phenolics may exhibit different bioavailability and release kinetics compared to free forms, potentially offering sustained antioxidant protection in food matrices or during gastrointestinal digestion.

The absence of detectable flavonoids in the HPLC analysis, despite measurable total flavonoid content by spectrophotometry, suggests that flavonoids are present predominantly as glycosides or other conjugated forms that were not efficiently extracted or detected under the chromatographic conditions used. Alternatively, the spectrophotometric method may overestimate flavonoid content due to interference from other phenolic compounds.

### 2.9. Microbiological Parameters of Nanocomposites

**Figure 6:**
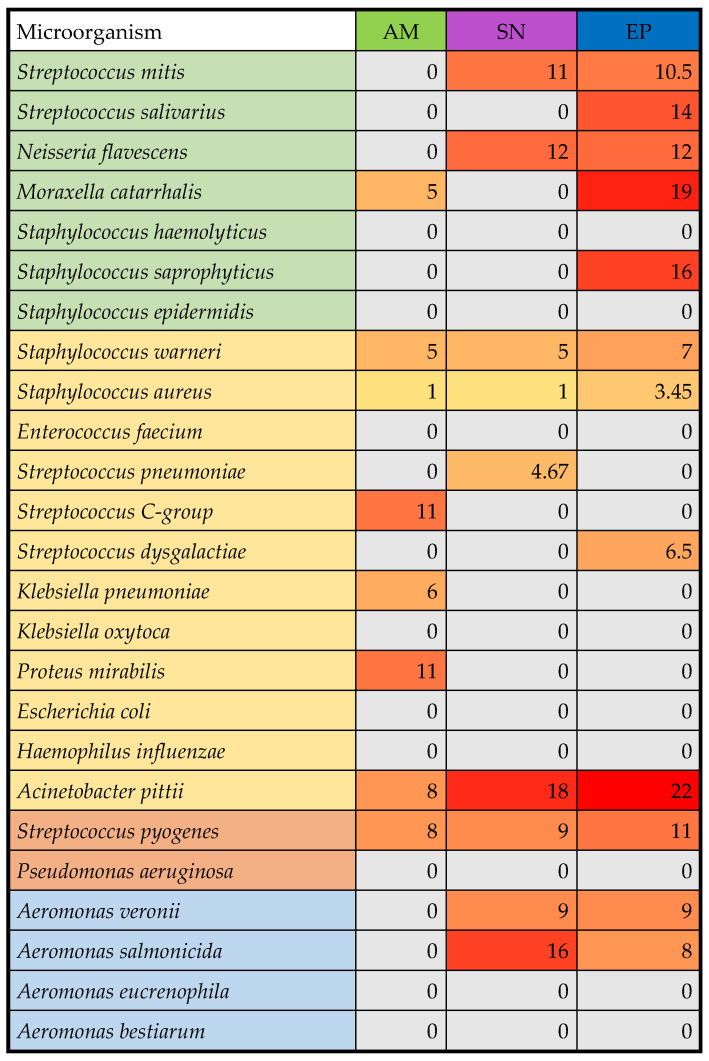
Antimicrobial activity of three lyophilized plant extracts encapsulated in psyllium/starch matrices against selected oral and opportunistic microorganisms. Color intensity reflects inhibition zone diameter (mm). Echinacea showed the broadest activity, elderberry was slightly more selective, and aronia demonstrated the smallest antibacterial potential. Rows are grouped by ecological role of microorganisms for comparison: oral commensals (greenish), opportunistic pathogens (yellow), true pathogens (reddish), and environmental species (blue). SN—biocomposites with *Sambucus nigra* (elderberry) extract; AM—biocomposites with *Aronia melanocarpa* (chokeberry fruit) extract; EP—biocomposites with *Echinacea purpurea* (purple coneflower) extract; PS—freeze-dried 4% potato starch gel.

**Figure 7:**
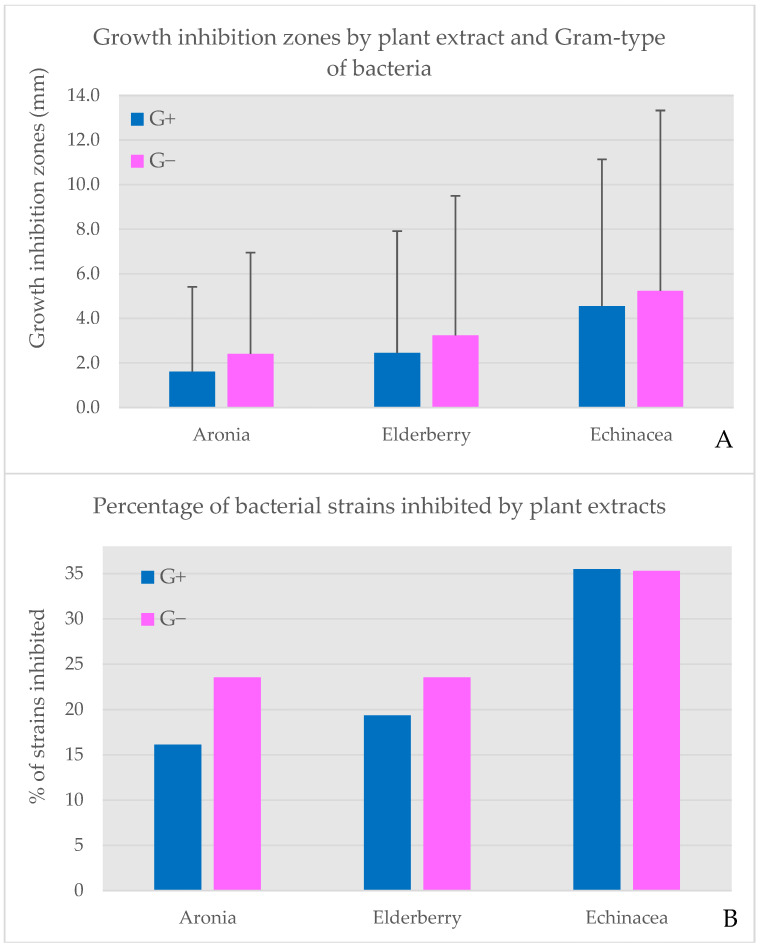
Comparative antimicrobial activity of three lyophilized plant extracts encapsulated in psyllium/starch matrices against Gram-positive (G+) and Gram-negative (G−) bacterial isolates. (**A**) shows the extract effectiveness expressed by the growth inhibition zone diameters (mm) with standard deviation. (**B**) shows the percentage of bacterial strains, the growth of which was inhibited by each extract. Echinacea exhibited the strongest overall activity, demonstrated by the highest growth inhibition and broadest spectrum of activity. Aronia showed minimal inhibition, suggesting microbiota-preserving potential.

Beyond antioxidant activity, the antimicrobial potential of the biocomposites was assessed against a panel of 48 bacterial isolates (Figure 6 and Figure 7; Supplementary Table S1) to evaluate their applicability as natural preservatives or functional ingredients targeting oral and opportunistic pathogens. The antimicrobial activity of three lyophilized biocomposites with plant extracts selected for this study (aronia, elderberry, and echinacea) was examined against a total of 48 bacterial isolates grouped by their role for comparison: oral commensals, opportunistic pathogens, true pathogens, and environmental species (blue) (Supplementary Table S1). Echinacea biocomposites (EP) demonstrated the broadest and most potent activity, followed by elderberry bio composites (SN) (more selective and weaker inhibition), while aronia biocomposites (AM) showed minimal effects (Figure 6 and Figure 7).

When analyzing the effects of the plant extracts against various groups of microorganisms, it could be clearly noticed that echinacea produced the largest and most frequent growth suppression against a variety of microbial groups. The mean growth inhibition zones observed for commensal bacteria *Moraxella catarrhalis* and *Staphylococus saprophyticus* and opportunistic pathogen *Acinetobacter pittii* exceeded 15 mm (Figure 6). Echinacea also inhibited the growth of more than 35% of both Gram-positive and Gram-negative bacteria (Figure 7). Elderberry inhibited a narrower subset of isolates (n = 10, i.e., 20.8% in total with a larger percentage of Gram-negatives affected). The largest growth inhibition zones were recorded for *Acinetobacter pittii* (opportunistic pathogen; 18 mm), followed by *Aeromonas salmonicida* (environmental bacterium, 16 mm) and a commensal *Neisseria flavescens* (12 mm). Aronia showed the smallest activity (both in terms of the growth inhibition zones and the percentage of strains affected), with measurable inhibition limited to a few (n = 8) isolates such as *Proteus mirabilis* and *Streptococcus* C-group. These patterns were clearly reflected in the heatmap, where echinacea formed a distinct cluster of high-intensity responses (Figure 6).

When inhibition zones were averaged by Gram reaction, a consistent trend also emerged. EP showed the highest mean inhibition for both Gram-positive (4.55 mm) and Gram-negative bacteria (5.24 mm), followed by SN (2.45 mm and 3.24 mm, respectively). AM remained the weakest composites, with mean zones below 3 mm for both groups. The bar chart summarizing these values highlights the clear separation between the composites and confirms the greater susceptibility of Gram-positive isolates (Figure 7A).

Finally, the percentage of strains inhibited by each biocomposites was calculated. EP inhibited over one-third of both Gram-positive (35.48%) and Gram-negative isolates (35.3%), demonstrating the broadest spectrum of activity. SN inhibited approximately one-fifth of Gram-positive isolates (19.35%) and a similar proportion of Gram-negative isolates (23.5%). AM showed the lowest coverage, inhibiting only 12.9% of Gram-positive and 23.5% of Gram-negative strains.

When combined with mean inhibition zones, these data reveal that echinacea is not only the most efficient extract but also the most broadly effective, whereas aronia remains largely microbiota-safe. These findings align with the known phytochemical composition of echinacea plant extracts, particularly caffeic acid derivatives, β-caryophyllene, α-pinene, germacrene D, α-phellandrene, β-pinene, γ-curcumene, and δ-cadinene, the antibacterial activity of which has been demonstrated in vitro [53]. In general, *Echinacea purpurea* has been repeatedly mentioned as an effective antimicrobial and immunomodulatory plant, hence its long history of medicinal use for a variety of conditions [10,54]. Oral and upper respiratory tract commensals (e.g., *Streptococcus mitis*, *Streptococcus salivarius*, *Moraxella catarrhalis*), the growth of which was inhibited by the examined extracts, are not typically targeted in antimicrobial and immunomodulatory formulations. However, their susceptibility to phenolic-rich plant extracts has been reported by, e.g., Karygianni et al. [55]. Such effects have been suggested as beneficial in terms of using natural phytochemicals as supplements or even substitutes for conventional agents in various biofilm-related diseases of oral cavity [55]. Importantly, all three plant extracts inhibited the growth of clinically relevant opportunistic pathogens, such as *Staphylococcus warneri*, *Staphylococcus aureus*, and *Acinetobacter pittii*, as well as a true pathogen—*Streptococcus pyogenes*. The latter is one of the major human pathogens that causes over 600 million infections and more than 500 thousand deaths annually [56], but it still remains highly susceptible to a variety of antimicrobial agents in vitro [56]. This bacterium has also been shown to be susceptible (next to *S. mutans*) to the plant-derived oils [57], which might be the cause of observations made in this study. The strong growth inhibition of *A. pittii* is also noteworthy, as members of this species have been reported as dangerous emergent pathogens, frequently carrying genes involved in a variety of resistance patterns, including carbapenem resistance [58].

Elderberry exhibited a narrower spectrum and smaller effectiveness of antibacterial activity than echinacea, but still its effect was significant against, e.g., *S. mitis*, *A. pittii*, and *A. salmonicida* (*p* < 0.05). In vitro antibacterial activity of elderberry extracts is generally determined by the content of phenolic acids and flavonoids. Importantly, the bioavailability of the active ingredients is among the factors that significantly affect the usefulness of elderberry extracts for health purposes [13]. The encapsulation of plant extracts developed in this study might contribute to increasing the bioavailability of active ingredients. Finally, aronia demonstrated the smallest antibacterial activity as compared to the previous two encapsulated plant extracts. Nevertheless, a number of previous studies (summarized by Ghanbari et al. [59]) demonstrated the antibacterial effects of *Aronia melanocarpa* active compounds (e.g., anthocyanins) against a wide range of pathogens and food spoilage bacteria.

Interestingly, when isolates were grouped by Gram reaction, all three extracts appeared to be more effective against Gram-negative than Gram-positive species (Figure 7A,B). There are numerous studies on the antibacterial activity of plant extracts and the observations on their effectiveness against bacteria of various Gram classification vary. However, more studies suggest that Gram-negative bacteria are less susceptible to various antimicrobial agents, due to their lipophilic outer membrane that could act as a natural barrier [59,60]. Plant-derived compounds interact differently with Gram-positive and Gram-negative bacteria, primarily due to the differences in their cell envelope structure. Gram-positive bacteria possess a thick, permeable peptidoglycan layer without an outer membrane, which allows phytochemicals to more easily penetrate the cell membrane. On the other hand, the cell wall of Gram-negative bacteria possesses an outer membrane with lipopolysaccharides that restricts its permeability [61]. Once the compounds reach the cell membrane, their activity does not differ between Gram-negative and Gram-positive bacteria. And so, gallic acid, detected only in SN fruit composites, disrupts cell membrane integrity, blocks catalase activity, and inhibits biofilm formation [62,63]. Protocatechuic acid, detected in SN and AM in similar amounts, enhances cell membrane permeability, reduces membrane potential, causes leakage of intracellular ions, leads to the accumulation of large amounts of reactive oxygen species, and inhibits the activity of enzymes: ATPase pyruvate kinase and succinate dehydrogenase [64]. Caffeic acid, the content of which was the highest in EP composites, followed by AM and SN, increases membrane permeability and causes leakage of intracellular nucleotides, while in Gram-negative bacteria, it also disintegrates the lipopolysaccharide layer of the outer membrane [65]. An increase in outer and plasma membrane permeability is also caused by *p*-coumaric acid, detected in SN, AM, and EP composites in the concentration of 5.1, 0.32, and 1.5 mg/100 g, respectively. It can also bind to the phosphate ion in the double helix of the DNA and intercalate the groove in DNA, affecting replication of proteins [66]. Finally, ferulic acid, detected only in EP composites, has been reported to increase membrane permeability by causing local rupture or pore formation [67]. In summary, all the compounds detected in the examined formulations increase the membrane permeability of both Gram-positive and Gram-negative bacteria, while some of them have additional properties, leading to the enhanced antibacterial effect. For this reason, it is not surprising that the order of increasing total concentration of phenolic acids in the plant extract composites (i.e., AM < SN < EP) appears to be directly reflected in their increasing antibacterial activity.

An important factor that might not only affect the overall antibacterial (as well as other) activity of active compounds from plant extracts, but also alter their antimicrobial performance and selectivity is their encapsulation in polymeric matrices. Several studies demonstrated that the antimicrobial activity of phenolic-rich plant extracts is generally enhanced or altered following their encapsulation in polysaccharide-based carriers. For example, Matouskova et al. [68] showed that herbal extracts packaged into polysaccharide particles and liposomes maintained strong inhibition against *Escherichia coli* (Gram-negative) and *Micrococcus luteus* (Gram-positive), with enhanced effects in some combinations, likely due to increased stability and sustained release of active compounds from the encapsulation matrix. Also as stated by Lima et al. [28], psyllium alone can exert some antimicrobial activity (although not observed in this study), but it becomes enhanced when combined with bioactive agents (e.g., essential oils or plant extracts), suggesting that it can act as a promising material for the development of innovative and active antibacterial formulations.

From an application perspective, the strong activity of *echinacea* against clinically relevant pathogens such as *Streptococcus pyogenes* and *Acinetobacter pittii*, combined with its high antioxidant capacity, positions it as a promising candidate for functional foods targeting oral health or immune support. The microbiota-safe profile of chokeberry, with minimal antimicrobial effects, may be advantageous for applications where preservation of beneficial bacteria is desired, such as in probiotic or prebiotic formulations.

## 3. Materials and Methods

### 3.1. Materials

Dry elderberry fruit (*Sambucus nigra* L.) was purchased from DARY NATURY Sp. z o.o. (Koryciny, Poland), dry chokeberry fruit (*Aronia melanocarpa*) from Dary Podlasia (Bielsk Podlaski, Poland), and dry purple coneflower (*Echinacea purpurea*) from Zioła z Doliny Bobru Grzywa Maciej (Wleń, Poland). Potato starch (PS) [Superior Standard] was purchased from PPZ Bronisław (Strzelno, Poland). The freeze-dried mucilage of psyllium (*Plantago psyllium*) was prepared according to Krystyjan et al. procedure [20]. Ethyl alcohol 96% p.a. grade was purchased from F.H.U. DOR-CHEM (Cracow, Poland) and used for extractions of bioactive compounds from plants. Grape seed oil from Monini was purchased in a neighborhood store.

### 3.2. Methods

#### 3.2.1. Extraction of Bioactive Compounds from Plants

The extraction process was experimentally optimized during preliminary studies in order to achieve the highest extraction efficiency. Dried elderberry fruits were ground into a fine powder using an RCMZ-800N grinder (Royal Catering, Berlin, Germany). Bioactive compounds were extracted from the plant material using a hydroalcoholic solvent system at a ratio of 10 g of ground dried plant material to 220 mL of solvent. The solvent was prepared by mixing ethanol (96%, p.a. grade) and distilled water in a volume ratio of 625 mL to 375 mL per 1 L of solution.

The mixture was subjected to ultrasonic treatment for 30 min in an ultrasonic bath (Sonic 10, Polsonic, Warsaw, Poland), followed by stirring on a magnetic stirrer at 600 rpm for 24 h. After extraction, the mixture was filtered through filter paper to remove the plant residue, and the solvent was subsequently removed under reduced pressure using a rotary evaporator (Heidolph Instruments GmbH & Co. KG, Schwabach, Germany) at 40 °C, yielding a dry extract.

The extraction yield was calculated as follows:

$$Y\;\left(\%\right)=\;\frac{m_{1}}{m_{2}}\times 100$$

*m*_1_—mass of the dry extract (g).

*m*_2_—mass of the raw material on a dry matter basis (g).

#### 3.2.2. Preparation of Nanocomposites

I. *Preparation of starch gel*

A 4% (*w*/*w*, based on dry matter) potato starch suspension was prepared and subsequently heated at 95 °C for 1 h under continuous stirring at 500 rpm using a magnetic stirrer (Heidolph RZR 2020, Heidolph Instruments GmbH & Co. KG, Germany). After heating, the gel was allowed to cool to room temperature. A portion of the gel was freeze-dried, while the remaining part was used for the preparation of nanocomposites.

II. *Preparation of Plantago psyllium mucilage gel*

A 0.5% (*w*/*w*, based on dry matter) suspension of freeze-dried Plantago psyllium mucilage (obtained according to the procedure described by Krystyjan et al. [20] was prepared by dissolving the material in distilled water at 70 °C for 30 min under continuous stirring at 500 rpm using a magnetic stirrer (Heidolph RZR 2020, Heidolph Instruments GmbH & Co. KG, Schwabach, Germany). Subsequently, the suspension was allowed to cool to room temperature.

III. *Preparation of nanocomposite:*

The encapsulation conditions were optimized in preliminary experiments. A mixture consisting of 5.0 g of dry extract, 5.0 g of grape seed oil, and 5.0 g of distilled water was homogenized using an ultrasonic homogenizer (20 kHz; Sonopuls HD 4200, Bandelin, Germany) for 5 min in continuous mode under cooling conditions (2–4 °C) using an ice-water bath. Cooling was applied to prevent temperature increase, which could otherwise lead to bioactive compound degradation and oil oxidation. Subsequently, 2 g of Plantago psyllium mucilage gel (0.5% *w*/*w*, based on dry matter) (prepared according to Section 3.2.2—II) was added dropwise using an automatic pipette, which resulted in a homogeneous nanoemulsion. In order to obtain the final nanocapsules, the nanoemulsion was slowly incorporated into 500 g of starch gel and homogenized (5 min, 12,000 rpm; Polytron PT 2500E homogenizer, Kinematica AG, Malters, Switzerland). The final material was lyophilized (freeze dryer Gamma 1–16 LSC, Christ, Osterode am Harz, Germany) to obtained microbiologically stable matrices.

Three variants of nanocomposites with plant extracts were prepared (Table 10):

SN—Biocomposites with *Sambucus nigra* (elderberry) extract.

AM—Biocomposites with *Aronia melanocarpa* (chokeberry fruit) extract.

EP—Biocomposites with *Echinacea purpurea* (purple coneflower) extract.

Additionally, for comparison purposes, a freeze-dried 4% potato starch gel was used:

PS—Freeze-dried 4% potato starch gel.

#### 3.2.3. Rheological Measurements

The rheological analysis was performed on the nanoemulsions and starch gel prior to their lyophilization based on the method described by Krystyjan et al. [25], with slight modifications in order to determine the rheological properties of the material. Measurements were performed using a RheoStress RS 6000 rotational rheometer (Thermo Scientific, Karlsruhe, Germany) equipped with a concentric cylinder CC26Ti system. Analyses were conducted on freshly prepared samples at 20.0 ± 0.1 °C, and each measurement was carried out in triplicate.

Flow curves: The shear rate was raised from 0.1 to 100 s^−1^, over a 5 min period and a subsequent decrease in shear rate from 100 to 0.1 s^−1^, over 5 min. Obtained flow curves are described by the Ostwald–de Waele rheological model:

$$\tau =K\cdot {\dot{\gamma }}^{n}$$

where *τ*—shear stress (Pa); *K*—consistency coefficient (Pa·s^n^); $\dot{\gamma }$—shear rate (s^−1^); *n*—flow behavior index.

#### 3.2.4. Scanning Electron Microscopy (SEM)

The size and morphology of the resulting nano/microcapsules were analyzed using a JEOL 7550 scanning electron microscope (Akishima, Tokyo, Japan) equipped with a secondary electron detector (SE). The imaging process was conducted at an accelerating voltage of 15 kV, employing a spot size of 1.0 nm. Prior to the collection of measurements, the samples were sputtered (K575X Turbo Sputter Coater) with 20 nm of chromium (Cr) to enhance the conductivity of the samples.

#### 3.2.5. FTIR Spectroscopy

FTIR spectra of the obtained films were measured using a Mattson 3000 FT-IR spectrophotometer equipped with a ReFractance 30SPEC 30-angle reflectance overlay and MIRacle ATR from PIKE Technologies Inc. (Madison, WI, USA). Measurements were made at 4 cm resolution, in the infrared region of 4000–750 cm^−1^. FTIR spectra underwent comprehensive processing, including baseline correction (automatic polynomial fitting), ATR absorption correction, and vector normalization. The aforementioned procedures were performed using Omnic 9 software (v9.12.1002, Thermo Fisher Scientific, Vacaville, CA, USA).

#### 3.2.6. Color Measurements

The color characteristics of the composites were evaluated using a Konica Minolta CM-3500d spectrophotometer (Konica Minolta Inc., Tokyo, Japan) fitted with a 30 mm measuring aperture. Measurements were carried out under standard illuminant D65 with a 10° observer angle. Color parameters were recorded according to the CIELAB color space, including L*, a*, and b* coordinates. The L* parameter describes the brightness of the sample, with values ranging from 0 (black) to 100 (white). The a* coordinate reflects the color position along the green–red axis, where negative values correspond to green tones and positive values indicate red tones. Similarly, the b* coordinate represents the blue–yellow axis, with negative values associated with blue hues and positive values with yellow hues. For each sample, five independent measurements were performed, and the results were expressed as mean values. On the basis of the primary color coordinates, additional color descriptors were calculated, including chroma (C*), hue angle (h*), and total color difference (∆E*). Chroma (C*) describes color saturation, reflecting the strength or vividness of the color in comparison to a neutral gray of identical lightness. Higher C* values indicate more intense and visually saturated colors and were calculated using the following equation:

$$C^{*}=\;\sqrt{{a^{*}}^{2}+{b^{*}}^{2}}$$

The hue angle (h*) defines the tonal position of a color within the color space, with characteristic reference angles of 0° for red, 90° for yellow, 180° for green, and 270° for blue. The hue angle was calculated as:

$$h^{*}={tan}^{-1}\left(\frac{b^{*}}{a^{*}}\right)$$

The total color difference (ΔE*) was used to quantify the magnitude of color changes between the analyzed samples and the control reference:

$${\Delta E}^{*}=\;\sqrt{{\Delta a}^{*2}+{\Delta b}^{*2}+\Delta L^{*2}}$$

#### 3.2.7. Thermal Analysis

Approximately 4 mg of the sample was weighed and sealed into aluminum pans. Subsequently, the samples were heated from 25 °C to 400 °C at a rate of 10 °C/min. The empty aluminum pan was used as a reference. The tests were performed with the DSC 204F1 Phoenix differential scanning calorimeter (Netzsch, Germany). The parameters of the observed thermal transition were calculated with Proteus Analysis software ver. 4.8.2 (Netzsch, Germany). The analyses were performed at last in two replication.

#### 3.2.8. Wettability and Free Surface Energy Determination

Contact angles and surface free energy values of the films were obtained using a Krüss DSA100M Drop Shape Analyzer (Krüss GmbH, Hamburg, Germany), which integrates an optical microscope with a high-speed digital camera operating at 20 fps. The instrument evaluates the droplet profile by fitting it to the Young–Laplace equation through an automated image-processing routine. Stainless steel needles (NE 44, Krüss GmbH; outer diameter: 0.5 mm) were used for dispensing the liquids. Droplet volumes were fixed at 11.5 mm^3^ for water and 2.5 mm^3^ for diiodomethane.

Dynamic contact angles were recorded for both water, representing a polar test liquid, and diiodomethane, which was used as a non-polar counterpart. Measurements were performed under controlled environmental conditions: the analysis chamber was thermostated using a circulating water bath, maintaining 22.0 ± 0.3 °C and constant humidity. For every film, at least five measurements were acquired, and results are presented as mean values with standard deviations.

The surface free energy of the films was calculated based on the Owens–Wendt approach [69] widely regarded as well-suited for polymeric surfaces. The same measurement procedure was used for the polar and non-polar liquids (water: δ = 72.30 mN/m, Millipore Q, 18.60 mΩ·cm; diiodomethane: δ = 50.80 mN/m).

#### 3.2.9. Polyphenols and Antioxidant Activity

*Procedure for the extraction of phenolic compounds from the analyzed fruit samples*

In order to extract the phenolics from the tested samples, approximately 10 g of the sample was weighed and transferred into a 100 mL volumetric flask. Flask was then filled with an aqueous–methanolic solution (80:20, *v*/*v*) to a volume of approximately 60 mL. The resulting mixture was protected from light and extracted on a mechanical shaker at room temperature for 24 h. After that period, volumetric flasks were filled up to 100 mL with the extraction solvent. The resulting extract was filtered and then defatted in a separatory funnel by triple extraction with petroleum ether. The obtained defatted extract was subsequently subjected to chromatographic analysis. To do so, the extract was purified earlier using solid-phase extraction (SPE). The SPE cartridge (Hypersil—Keystone, Thermo Scientific, Bellefonte, PA, USA) packed with a reversed phase (RP-18) sorbent (1 g filling mass and 6 mL column volume) was firstly conditioned with 5 mL of methanol. Then, 3 mL of the analyzed extract was applied to the SPE cartridge and eluted using methanol into a 10 mL volumetric flask to make up the volume to 10 mL. The obtained purified extract was directly subjected to both chromatographic and spectrophotometric analyzes.

*Determination of total phenolic content in the analyzed samples using Folin method*

Total phenolic content (TPC) in the samples under study was determined using Folin–Ciocalteu’s method developed by Singleton and Rossi [70]. For this aim, 0.5 mL of the aqueous–methanolic extract was mixed in a tube with 2.5 mL of appropriately diluted Folin–Ciocalteu’s reagent. After a few min. of incubation, 2 mL of a 7.5% (*m*/*v*) Na_2_CO_3_ solution was added to each tube. The blank sample was prepared in the same way, using deionized water instead of extract. After 2 h of incubation, the absorbance of the solution was measured using an UV/Vis spectrophotometer (Jasco, Tokyo, Japan) against a blank sample. The obtained results were expressed in mg of gallic acid per 100 g of the sample, calculated as the mean of the three analytical replicates. The calculations were based on the calibration curve based on gallic acid solutions in the concentration range from 20 to 200 mg/L.

*Determination of total flavonoid content in the tested samples*

Total flavonoid content (TFC) in the samples under study was determined using the method developed by Barnum et al. [71]. To do so, 1 mL of the aqueous–methanolic extract was mixed in a tube with 4 mL of deionized water. Subsequently, 0.3 mL of a 15% NaNO_2_ solution following by 0.3 mL of a 10% AlCl_3_ methanolic solution, and finally 4 mL of a 4% (m/v) NaOH solution was added, with the contents mixed after each reagent addition. The final volume of reaction mixture was then adjusted to 10 mL with deionized water.

A blank sample was prepared in the same way, replacing the extract with deionized water. The obtained results were expressed in quercetin equivalents (mg) per 100 g of the sample, calculated as the mean of the three analytical repetitions. The calibration curve was based on quercetin solutions in the concentration range from 0.1 to 1 g/L.

*Determination of antioxidant activity using a DPPH assay of the analyzed samples*

Antioxidant activity (AA) of the samples under study was determined using the method developed by Blois [72]. To do so, 0.1 mL of the aqueous–methanolic extract was mixed in a tube with 3.0 mL of 0.1 mM methanolic DPPH radical solution. After 60 min of incubation, an absorbance of resulting solution was measured at λ = 515 nm using an UV/Vis spectrophotometer (Jasco, Tokyo, Japan) against methanol. The obtained results were expressed in millimoles of Trolox per 100 g of sample, calculated as the mean of the three analytical replicates. The calculations were based on the calibration curve, which was made using Trolox solutions in the concentration range from 0.1 to 1 mM/L.

*Determination of antioxidant activity using an ABTS assay of the analyzed samples*

The antioxidant activity (AA) of the analyzed samples under study was determined using the method developed by Baltrušaitytė et al. [73]. To do so, 0.1 mL of the aqueous–methanolic extract was mixed in a tube with 6.0 mL of ABTS cation radical solution, appropriately diluted with the phosphate buffer (pH = 7.4). After 30 min of incubation, an absorbance of the resulting solution was measured at λ = 734 nm using an UV/Vis spectrophotometer (Jasco, Tokyo, Japan) against the phosphate buffer. The obtained results were expressed in millimoles of Trolox per 100 g of sample, calculated as the mean of the three analytical replicates. The calculations were based on the calibration curve, which was made using Trolox solutions in the concentration range from 0.1 to 1 mM/L.

*Determination of reducing activity of the samples using a FRAP assay*

The reducing activity of the samples under study was determined using the method developed by Benzie and Strain [74]. To do so, 3.3 mL of acetate buffer (pH = 3.6) was mixed in a tube with 0.33 mL of 20 mM FeCl_3_ solution, followed by 0.33 mL of 10 mM TPTZ (2,4,6-tris(2-pyridyl)-1,3,5-triazine) solution in 40 mM HCl acid and finally was incubated at the temp. of 37 °C. After incubation, 0.33 mL of the extract was added to the obtained mixture followed by the next incubation at a temperature of 37 °C for 15 min. After cooling, the absorbance of the resulting mixture was measured at λ = 593 nm using an UV/Vis spectrophotometer (Jasco, Tokyo, Japan) against the blank sample including pure water instead of the extract itself. The results were expressed as micromoles of Fe^+2^ ions per 100 g of sample, calculated as the mean of the three analytical replicates. The calculations were based on the calibration curve equation, which was made using FeSO_4_ solutions in the concentration range from 200 to 1200 µM/L.

*Procedure for the analysis of free phenolic compounds in the tested samples using the chromatographic method*

The chromatographic analysis of extracts under study was carried out using a high-performance liquid chromatograph (Jasco, Tokyo, Japan) equipped with a gradient pump and diode array detector (DAD, Jasco, Japan). The temp. of the column was maintained at 30 °C, and the injection volume was 20 µL. The presence of hydroxybenzoic acids (i.e., protocatechuic acid) was identified at 280 nm, and hydroxycinnamic acids (i.e., caffeic, p-coumaric, ferulic acids) were identified at 320 nm, whereas flavonoids (i.e., catechin and hesperidin) were identified at a λ = 320 nm. Chromatographic analysis was carried out using a reversed phase (RP-18) column (Purospher, 250 × 4.6 mm, 5 µm particle size; Merck, Darmstadt, Germany), with the solvent flow rate maintained at 1 mL/min. The gradient elution chromatography was employed using the two mobile phases: phase A (2.5%, (m/v), acetic acid solution in water) and phase B (acetonitrile of gradient grade), based on the earlier described procedure [75]. The chromatographic analysis was carried out as follows: during the first 10 min, a linear gradient was applied, followed by an increase in the proportion of phase B to 15, 20, 30, and 40% at 20, 30, 40, and 50 min, respectively. After the analysis, the chromatographic column was flushed isocratically with pure acetonitrile for 10 min prior to the next run.

Qualitative analysis was made by comparing the retention times of appropriate chromatographic peaks with those of phenolic standards purchased from Sigma (Steinheim, Germany), as well as by comparing the absorption spectra of detected peaks with the corresponding spectra of the phenolic standards.

Quantitative analysis was made using the calibration curves prepared for the analyzed phenolics, based on the solutions within the concentration range of 0.02–0.2 mg/mL. The chromatographic measurements were performed in triplicate for both the analyzed samples and the phenolic standards. For the calibration curves, the concentration values were calculated as the means of three repetitions, whereas phenolic contents in the tested samples were expressed as the mean of the three replicate analyses.

*Procedure for Extraction and Analysis of Bound and Free Forms of Phenolic Compounds in the Tested Samples Using a Chromatographic Method*

In order to analyze the presence and level of phenolics occurring in bound forms (i.e., as esters and glycosides) in the tested samples, the aqueous–methanolic extracts were subjected to an alkaline hydrolysis according to the procedure developed by Nardini and Ghiselli [76]. To do so, 10 mL of the aqueous–methanolic extract was mixed with 90 mL of a hydrolyzing solution (containing 1% of ascorbic acid and 10 mM EDTA dissolved in 2 M NaOH solution). The resulting solution was incubated at 30 °C under an inert gas (argon). After completion of the alkaline hydrolysis, the solution was cooled to room temperature and subsequently acidified with diluted HCl acid (1:2, *v*/*v*) until pH = 2 was reached.

The extraction of phenolics released from their bounded forms was performed according to the procedure described previously by Socha et al. [77]. To do so, the previously acidified phenolic solution was saturated with NaCl until saturation was reached. Subsequently, the obtained solution was extracted three times with ethyl acetate in a separatory funnel. After the extraction, the collected ethyl acetate fractions were combined and evaporated using a vacuum rotary evaporator at 39 °C until a dry residue was obtained. The resulting solid residue was dissolved in 5 mL of methanol and then transferred to a polypropylene tube and stored at 5 °C until the chromatographic analysis. Before HPLC analysis, the prepared extracts were purified by solid phase extraction (SPE), analogously to the procedure applied earlier for the samples not subjected to alkaline hydrolysis.

#### 3.2.10. Microbiological Analysis

Lyophilized plant extracts (nanocomposites) were supplied in dry powder form. Due to their limited solubility in aqueous solvents, each extract was suspended in sterile distilled water to obtain a semi-solid, paste-like matrix suitable for antimicrobial testing. Approximately 0.5 mg of extract was transferred into a 1.5 mL microcentrifuge tube, followed by the addition of 500 µL of sterile water. Samples were vortexed until a homogeneous, thick suspension was formed. All suspensions were freshly prepared on the day of analysis.

Bacterial strains used in the analysis comprised 48 isolates, including physiological, commensal species, e.g., *Streptococcus salivarius*, *Streptococcus mitis*, *Moraxella catarrhalis*, or *Neisseria flavescens*; opportunistic pathogens of oral cavity or airways, e.g., *Haemophilus influenzae*, *Streptococcus* C-group, and *Staphylococcus aureus*; and true pathogens of oral cavity/airways, i.e., *Streptococcus pyogenes* and *Pseudomonas aeruginosa*. Environmental strains of, e.g., *Aeromonas* spp., were used for comparison. Bacterial suspensions were adjusted to 0.5 McFarland standard and spread evenly on Mueller–Hinton Agar (MHA) plates using sterile cotton swabs to obtain confluent growth.

Because the extracts formed a viscous, non-diffusible suspension that could not be reliably pipetted into agar wells or onto filter disks, their antimicrobial activity was first evaluated using a modified agar surface application method. After inoculating the MHA plates, a loopful of each extract suspension was aseptically transferred onto the agar surface. The material was gently shaped to form a compact spot to minimize its spreading over the plate. Plates were first incubated at room temperature for 24 h to allow liberation of extracts from the capsules, followed by incubation at 36 ± 1 °C for another 24 h.

After the incubation period, the presence or absence of clear or reduced-growth zones surrounding the extract spots was searched for. In the case of its presence, the diameter of the growth inhibition zone (including the application spot) was measured in millimeters. To complement the above approach and to evaluate antimicrobial activity under standardized diffusion conditions, disk diffusion assay was performed.

Sterile 6 mm paper disks (Oxoid or equivalent) were placed on a sterile Petri plate and loaded with 20 µL of the plant extract suspension using a micropipette. Disks were allowed to air-dry until the material was fully absorber. Such prepared disks were placed on freshly inoculated MHA plates. Controls included a blank disk loaded with starch suspension and a blank disk loaded with sterile water. Plates were incubated in the same conditions as in the case of the modified agar surface application method. After incubation, growth inhibition zones were measured and expressed in millimeters. All tests for both methods were performed in triplicate. Inhibition zone diameters were presented as mean ± standard deviation.

#### 3.2.11. Statistical Analysis

The data were subjected to statistical analysis using one-way analysis of variance (ANOVA) followed by Fisher’s test (*p* < 0.05) to determine significant differences between the tested samples.

## 4. Conclusions

The starch–psyllium binary matrix successfully encapsulated *Sambucus nigra*, *Aronia melanocarpa*, and *Echinacea purpurea* extracts, creating porous sponge-like structures containing uniformly distributed spherical particles measuring 800–1500 nm. Scanning electron microscopy confirmed the homogeneous integration of extract-loaded oil droplets within the polysaccharide network, while Fourier-transform infrared spectroscopy revealed molecular-level interactions including hydrogen bonding between polysaccharide hydroxyl groups and phenolic compounds, alongside hydrophobic associations with grape seed oil triglycerides. These structural modifications translated into enhanced functional properties relevant for food applications. The nanoemulsions exhibited pseudoplastic shear-thinning behavior with low consistency indices, facilitating industrial processing. Lyophilized composites preserved extract-specific color profiles clearly distinguishable to the human eye, demonstrating effective pigment immobilization critical for consumer acceptance of fortified products. Thermal analysis indicated extract-dependent effects on matrix behavior. The aronia extract acted as a plasticizer, lowering glass transition temperature by 20 °C through polyphenol–starch interactions that increased chain mobility. All extracts delayed thermal degradation onset, suggesting polyphenol-mediated stabilization of the polysaccharide backbone during processing and storage. Surface characterization revealed significantly increased hydrophobicity, particularly evident in reduced polar contributions to surface free energy, which positions these materials as potential moisture barriers for food packaging applications. Bioactivity closely followed extract phytochemical profiles. Antioxidant capacity showed strong correlation with total phenolic content, driven primarily by caffeic acid in echinacea systems. Antimicrobial performance varied distinctly: echinacea demonstrated broad-spectrum Gram-positive and Gram-negative inhibition, elderberry showed selective activity, and chokeberry effects remained minimal. Enhanced Gram-negative sensitivity suggests improved phenolic availability and membrane interactions following encapsulation.

Collectively, these findings validate the starch–psyllium system as a versatile, food-grade platform for stabilizing and delivering bioactive plant extracts. The matrix enables tunable functionality through extract selection while maintaining processability and structural integrity, warranting further investigation into controlled release profiles, sensory integration, and in vivo bioavailability for functional food development.

## Figures and Tables

**Table 1:** Extraction yield of plant extracts encapsulated in psyllium/starch matrices.

Raw Material	Extraction Yield (%)
*Sambucus nigra*	59.12 ± 0.54 ^a^
*Aronia melanocarpa*	53.67 ± 0.65 ^b^
*Echinacea purpurea*	42.39 ± 0.77 ^c^

The values are expressed as the mean ± standard deviation. The presence of the same superscript letter (a, b, c) in each column indicates that there is no statistically significant difference between the values (*p* < 0.05).

**Table 2:** Parameters of the Ostwald–de Waele rheological model and hysteresis area of plant extracts encapsulated in psyllium/starch matrices.

Sample of Emulsion	Ostwald–de Waele Rheological Model			Area of Hysteresis Loop (Pa/s)
	K (Pa/s^n^)	n (-)	R^2^	
PS	1.90 ± 0.00 ^a^	0.96 ± 0.00 ^a^	0.9979	1700.67 ± 193.00 ^a^
SN	0.21 ± 0.01 ^c^	0.78 ± 0.01 ^c^	0.9999	16.62 ± 1.21 ^b^
AM	0.25 ± 0.01 ^b^	0.77 ± 0.01 ^d^	0.9999	19.28 ± 1.03 ^b^
EP	0.18 ± 0.01 ^d^	0.79 ± 0.00 ^b^	0.9998	18.01 ± 0.96 ^b^

K—consistency coefficient; n—flow behavior index; R^2^—the coefficient of determination. The values are expressed as the mean ± standard deviation. The presence of the same superscript letter (a, b, c, d) in each column indicates that there is no statistically significant difference between the values (*p* < 0.05). SN—biocomposites with *Sambucus nigra* (elderberry) extract; AM—biocomposites with *Aronia melanocarpa* (chokeberry fruit) extract; EP—biocomposites with *Echinacea purpurea* (purple coneflower) extract; PS—freeze-dried 4% potato starch gel.

**Table 3:** Color parameters of plant extracts encapsulated in psyllium/starch matrices.

Composites	L* (D65)	a* (D65)	b* (D65)	C*	h*	ΔE*
PS	97.34 ± 0.10 ^a^	−0.13 ± 0.01 ^d^	2.17 ± 0.01 ^d^	2.18 ± 0.01 ^d^	178.49 ± 0.01 ^b^	-
SN	76.25 ± 0.23 ^d^	13.52 ± 0.12 ^a^	6.60 ± 0.04 ^b^	15.05 ± 0.13 ^b^	0.45 ± 0.00 ^d^	25.52
AM	86.23 ± 0.64 ^c^	7.70 ± 0.33 ^b^	3.90 ± 0.08 ^c^	8.63 ± 0.33 ^c^	0.47 ± 0.01 ^c^	13.70
EP	90.59 ± 0.42 ^b^	−1.14 ± 0.03 ^c^	16.69 ± 0.23 ^a^	16.73 ± 0.23 ^a^	178.50 ± 0.00 ^a^	16.04

The values are expressed as the mean ± standard deviation. The presence of the same superscript letter (a, b, c, d) in each column indicates that there is no statistically significant difference between the values (*p* < 0.05). SN—biocomposites with *Sambucus nigra* (elderberry) extract; AM—biocomposites with *Aronia melanocarpa* (chokeberry fruit) extract; EP—biocomposites with *Echinacea purpurea* (purple coneflower) extract; PS—freeze-dried 4% potato starch gel.

**Table 4:** Melting peak parameters of plant extracts encapsulated in psyllium/starch matrices.

Sample	T_onm_	T_pm_	T_endm_	−ΔH_m_	T_pex_
	°C	°C	°C	J·g^−1^	°C
PS	209.1 ± 20.9 ^a^	213.8 ± 20.8 ^a^	220.4 ± 20.6 ^a^	59.0 ± 8.0 ^a^	311.6 ± 2.5 ^b^
SN	nd	nd	nd	nd	311.0 ± 0.8 ^b^
AM	nd	nd	nd	nd	329.4 ± 1.6 ^c^
EP	143.0 ± 23.5 ^a^	156.6 ± 20.4 ^a^	184.2 ± 12.2 ^a^	53.1 ± 2.1 ^a^	302.5 ± 1.7 ^a^
One-way ANOVA—*p*	0.097	0.11	0.166	0.421	0.001

The values are expressed as the mean ± standard deviation. The presence of the same superscript letter (a, b, c) in each column indicates that there is no statistically significant difference between the values (*p* < 0.05). nd—not detected. SN—biocomposites with *Sambucus nigra* (elderberry) extract; AM—biocomposites with *Aronia melanocarpa* (chokeberry fruit) extract; EP—biocomposites with *Echinacea purpurea* (purple coneflower) extract; PS—freeze-dried 4% potato starch gel.

**Table 5:** Glass transition characteristic of plant extracts encapsulated in psyllium/starch matrices.

Sample	Tong	Tmidg	Tinfg	Tendg	−Δc_p_
	°C	°C	°C	°C	°C
PS	102.0 ± 20.9 ^a^	108.5 ± 20.9 ^a^	109.2 ± 20.9 ^a^	112.8 ± 20.9 ^a^	0.377 ± 0.120 ^a^
SN	91.3 ± 20.9 ^a^	117.4 ± 20.9 ^a^	112.3 ± 20.9 ^a^	127.4 ± 20.9 ^a^	0.764 ± 0.120 ^c^
AM	81.3 ± 20.9 ^a^	103.0 ± 20.9 ^a^	97.0 ± 20.9 ^a^	114.6 ± 20.9 ^a^	0.580 ± 0.120 ^b^
EP	101.1 ± 20.9 ^a^	109.5 ± 20.9 ^a^	110.5 ± 20.9 ^a^	115.4 ± 20.9 ^a^	0.351 ± 0.120 ^a^
One-way ANOVA—*p*	0.127	0.483	0.705	0.477	0.007

The values are expressed as the mean ± standard deviation. The presence of the same superscript letter (a, b, c) in each column indicates that there is no statistically significant difference between the values (*p* < 0.05). SN—biocomposites with *Sambucus nigra* (elderberry) extract; AM—biocomposites with *Aronia melanocarpa* (chokeberry fruit) extract; EP—biocomposites with *Echinacea purpurea* (purple coneflower) extract; PS—freeze-dried 4% potato starch gel.

**Table 6:** Water and diiodomethane contact angle values of the starch control matrix (PS) and nanocomposites containing plant extracts: *Sambucus nigra* (SN), *Aronia melanocarpa* (AM), and *Echinacea purpurea* (EP).

Sample	Water [°]	Diiodomethane [°]
PS	84.4	41.2
SN	90.1	50.3
AM	89.0	40.0
EP	81.9	44.8

**Table 7:** Surface free energy of plant extracts encapsulated in psyllium/starch matrices.

Sample	Dispersive Energy [mJ/m^2^]	Polar Energy [mJ/m^2^]	Energy Total [mJ/m^2^]
PS	41.59	1.91	43.50
SN	36.86	1.26	38.12
AM	43.89	0.74	44.63
EP	38.34	3.14	41.47

**Table 8:** Total polyphenol and flavonoid content and antioxidant and reducing activity of plant extracts encapsulated in psyllium/starch matrices.

Sample	Total Phenolic Content mg GAE/100 g	Total Flavonoids Content mg QE/100 g	DPPH Assay mM TE/100 g	ABTS Assay mM TE/100 g	FRAP Assay mM Fe (II)/100 g
PS	0.00	0.00	0.00	0.00	0.644 ^d^ ±0.016
SN	568.80 ^b^ ±13.33	1.007 ^b^ ±0.091	2.853 ^b^ ±0.058	7.304 ^b^ ±0.451	8.502 ^b^ ±0.372
AM	261.27 ^c^ ±5.63	0.473 ^c^ ±0.032	2.209 ^c^ ±0.071	3.343 ^c^ ±0.112	4.370 ^c^ ±0.219
EP	921.74 ^a^ ±16.25	4.268 ^a^ ±0.969	6.517 ^a^ ±0.104	9.308 ^a^ ±0.496	15.296 ^a^ ±0.519

The values are expressed as the mean ± standard deviation. The presence of the same superscript letter (a, b, c, d) in each column indicates that there is no statistically significant difference between the values (*p* < 0.05). GAE—gallic acid equivalents; QE—quercetin equivalents; TE—Trolox equivalents; Fe (II)—ferrous ions equivalents; mM—milimoles. SN—biocomposites with *Sambucus nigra* (elderberry) extract; AM—biocomposites with *Aronia melanocarpa* (chokeberry fruit) extract; EP—biocomposites with *Echinacea purpurea* (purple coneflower) extract; PS—freeze-dried 4% potato starch gel.

**Table 9:** Content of phenolic acids present in free and bound forms determined by chromatographic analysis of plant extracts encapsulated in psyllium/starch matrices.

Samples	Gallic Acid	Protocatechuic Acid	Caffeic Acid	p-Coumaric Acid	Ferulic Acid
	Content of phenolic compounds in the tested samples in free form (analyzed before an alkaline hydrolysis) mg/100 g				
PS	—	—	—	—	—
SN	0.525 ± 0.005	0.711 ± 0.035	—	—	—
AM	—	—	—	—	—
EP	—	—	—	—	—
Total phenolic acid content in the samples (mg/100 g) (including the sum of free and bound phenolic compounds)					
PS	—	—	—	—	—
SN	0.525 ± 0.005	1.063 ^b^ ± 0.010	0.782 ^c^ ± 0.036	5.111 ^a^ ± 0.021	—
AM	—	1.181 ^a^ ± 0.049	5.234 ^b^ ± 0.022	0.324 ^c^ ± 0.004	—
EP	—	—	49.493 ^a^ ± 1.101	1.545 ^b^ ± 0.020	2.522 ± 0.012

The values are expressed as the mean ± standard deviation. The presence of the same superscript letter (a, b, c) in each column indicates that there is no statistically significant difference between the values (*p* < 0.05). SN—biocomposites with *Sambucus nigra* (elderberry) extract; AM—biocomposites with *Aronia melanocarpa* (chokeberry fruit) extract; EP—biocomposites with *Echinacea purpurea* (purple coneflower) extract; PS—freeze-dried 4% potato starch gel.

**Table 10:** The study materials: raw materials, emulsions of plant extracts encapsulated in psyllium/starch matrices and biocomposites of plant extracts encapsulated in psyllium/starch matrices.

Form of Sample	Raw Material	Nanoemulsion	Biocomposites (Freeze-Dried Nanoemulsions)
*Sambucus nigra*	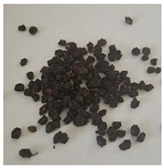	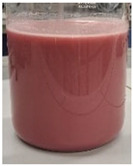	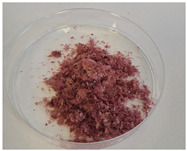
*Aronia* *melanocarpa*	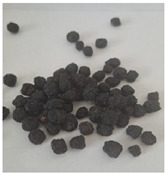	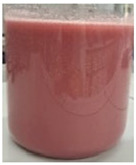	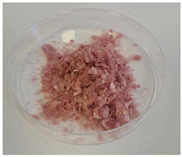
*Echinacea* *purpurea*	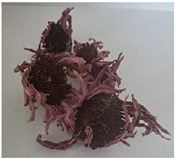	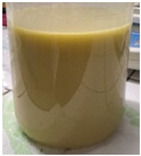	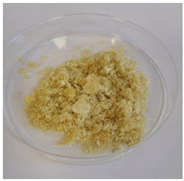

## Data Availability

The data presented in this study are available on request from the corresponding author. The data are not publicly available due to privacy restrictions and ongoing research utilizing the same dataset.

## Supplementary Materials

The following supporting information can be downloaded at: https://www.mdpi.com/article/10.3390/molecules31061026/s1, Table S1: Antimicrobial activity of biocomposites containing plant extracts against Gram-positive and Gram-negative bacteria.

## Abbreviations

The following abbreviations are used in this manuscript:

SEM	Scanning Electron Microscopy
FTIR	Fourier-Transform Infrared Spectroscopy
CIE Lab*	Commission Internationale de l’Éclairage Lightness and Color Coordinates
TPC	Total Polyphenol Content
TFC	Total Flavonoid Content
HPLC	High-Performance Liquid Chromatography
AA	Antioxidant Activity
GAE	Gallic Acid Equivalents
QE	Quercetin Equivalents
TE	Trolox Equivalents
FRAP	Ferric ion Reducing Antioxidant Parameter
ABTS	2,2′-azinobis(3-ethylbenzothiazoline-6-sulfonic acid) radical cation decolorization assay
DPPH	2,2-diphenyl-1-picrylhydrazyl radical scavenging assay
DSC	Differential Scanning Calorimetry
SFE	Surface Free Energy
