# Supplementary material for: Encapsulation of Plant Extracts in a Psyllium/Starch Matrix: Synthesis and Functional Properties
Source: Molecules. 2026 Mar 19;31(6):1026. doi: 10.3390/molecules31061026 (PMC13029679; doi:10.3390/molecules31061026)
Supplement: Supplementary file 1 [file molecules-31-01026-s001.zip › molecules-4177587-supplementary.pdf]

Supplementary

Table S1.

Gram	Identification	Control	SN	AM	EP
G+	Streptococcus mitis	0	22	0	21
G+	Streptococcus mitis	0	0	0	0
G+	Streptococcus salivarius	0	0	0	14
G+	Staphylococcus haemolyticus	0	0	0	0
G+	Staphylococcus epidermidis	0	0	0	0
G+	Staphylococcus epidermidis	0	0	0	0
G+	Staphylococcus epidermidis	0	0	0	0
G+	Staphylococcus saprophyticus	0	0	0	16
G+	Staphylococcus warneri	0	10	12	11
G+	Staphylococcus warneri	0	10	0	9
G+	Staphylococcus warneri	0	0	0	0
G+	Staphylococcus warneri	0	0	8	8
G+	Staphylococcus aureus	0	0	0	14
G+	Staphylococcus aureus	0	0	0	14
G+	Staphylococcus aureus	0	0	0	0
G+	Staphylococcus aureus	0	0	0	0
G+	Staphylococcus aureus	0	0	11	0
G+	Staphylococcus aureus	0	0	0	0
G+	Staphylococcus aureus	0	0	0	0
G+	Staphylococcus aureus	0	0	0	0
G+	Staphylococcus aureus	0	0	0	0
G+	Staphylococcus aureus	0	0	0	0
G+	Staphylococcus aureus	0	11	0	10
G+	Enterococcus faecium	0	0	0	0
G+	Streptococcus pneumoniae	0	0	0	0
G+	Streptococcus pneumoniae	0	0	0	0
G+	Streptococcus pneumoniae	0	14	0	0
G+	Streptococcus C-group	0	0	11	0
G+	Streptococcus dysgalactiae	0	0	0	13
G+	Streptococcus dysgalactiae	0	0	0	0
G+	Streptococcus pyogenes	0	9	8	11
G-	Neisseria flavescens	0	12	0	12
G-	Moraxella catarrhalis	0	0	10	16
G-	Moraxella catarrhalis	0	0	0	22
G-	Klebsiella pneumoniae	0	0	0	0
G-	Klebsiella oxytoca	0	0	0	0

G-	Klebsiella pneumoniae	0	0	12	0
G-	Proteus mirabilis	0	0	11	0
G-	Escherichia coli	0	0	0	0
G-	Haemophilus influenzae	0	0	0	0
G-	Acinetobacter pittii	0	18	8	22
G-	Pseudomonas aeruginosa	0	0	0	0
G-	Aeromonas veronii	0	9	0	9
G-	Aeromonas salmonicida	0	16	0	8
G-	Aeromonas eucrenophila	0	0	0	0
G-	Aeromonas eucrenophila	0	0	0	0
G-	Aeromonas bestiarum	0	0	0	0
G-	Aeromonas bestiarum	0	0	0	0
	mean	0	2.73	1.90	4.79
	SD	0	5.70	4.05	7.07

SN – biocomposites with *Sambucus nigra* (elderberry) extract, AM – biocomposites with *Aronia melanocarpa* (chokeberry fruit) extract, EP – biocomposites with *Echinacea purpurea* (purple coneflower) extract, PS – freeze-dried 4% potato starch gel.
